# Supplementary material for: Identification of photoperiod-induced specific miRNAs in the adrenal glands of Sunite sheep (Ovis aries)
Source: Front Vet Sci. 2022 Jul 22;9:888207. doi: 10.3389/fvets.2022.888207 (PMC9354845; doi:10.3389/fvets.2022.888207)
Supplement: Supplementary file 1 [file Data_Sheet_1.ZIP › Supplementary file/miRDeep2 file.zip!/miRDeep2 file/Novel_Structure/LP42A3/Novel_94.pdf]

Provisional ID	:	NC_019484.2_24819
Score total	:	10.5
Score for star read(s)	:	3.9
Score for read counts	:	3.2
Score for mfe	:	1.9
Score for randfold	:	1.6
Score for cons. seed	:	
Total read count	:	18
Mature read count	:	16
Loop read count	:	0
Star read count	:	2

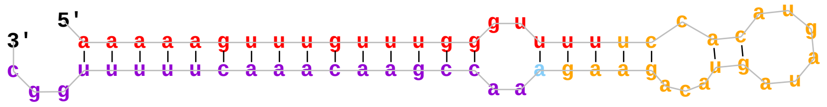

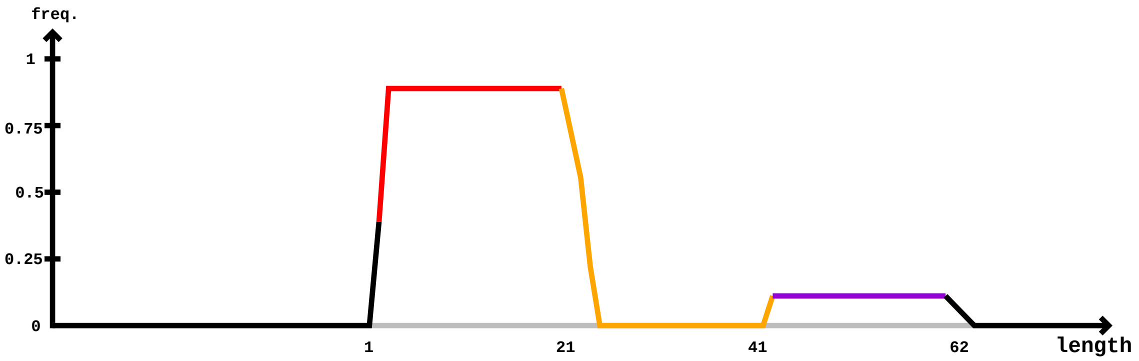

Mature Star

```
5'- gggugaggaacucgguacuauugggguuggcaaaaaaguuuguuuggguuuuuccacaugauaguacagaagaaaccgaacaaacuuuuuggcuaauccaauagcagc   -3'   obs
    gggugaggaacucgguacuauugggguuggcaaaaaaguuuguuuggguuuuuccacaugauaguacagaagaaaccgaacaaacuuuuuggcuaauccaauagcagc         exp
    ((((.....))))....((((((((.(((((.(((((((((((((((..(((((.((......))...)))))..)))))))))))))))).)))))))))))))....    reads   mm   sample
    ................................aaaaaguuuguuCggguuuu........................................................    4       1    seq
    ................................aaaaGguuuguuuggguuuuuc......................................................    1       1    seq
    ................................aaaaaguuuguuuggguuuuucc.....................................................    2       0    seq
    .................................Gaaaguuuguuuggguuuuu.......................................................    1       1    seq
    .................................aaaGguuuguuuggguuuuu.......................................................    1       1    seq
    .................................Gaaaguuuguuuggguuuuuc......................................................    2       1    seq
    .................................aaaaguuuguuuggguuuuuU......................................................    1       1    seq
    .................................aaaGguuuguuuggguuuuuc......................................................    1       1    seq
    .................................aCaaguuuguuuggguuuuuc......................................................    1       1    seq
    .................................Gaaaguuuguuuggguuuuucc.....................................................    2       1    seq
    .........................................................................aacUgaacaaacuuuuuggc...............    1       1    seq
    .........................................................................aUccgaacaaacuuuuuggc...............    1       1    seq
```
